# Testosterone and occupational burnout in professional male firefighters

**DOI:** 10.1186/s12889-021-10446-z

**Published:** 2021-02-23

**Authors:** Denis Vinnikov, Zhanna Romanova, Gulnara Kapanova, Aizhan Raushanova, Sundetgali Kalmakhanov, Alexander Zhigalin

**Affiliations:** 1 al-Farabi Kazakh National University, 71 al-Farabi avenue, Almaty, Kazakhstan 050040; 2 Peoples’ Friendship University of Russia (RUDN University), 6 Miklukho-Maklaya street, Moscow, Russian Federation 117198; 3 National Research Tomsk State University, 36 Lenin avenue, Tomsk, Russian Federation 634050; 4 Federal Research Center of Fundamental and Translational Medicine, 2 Timakov Street, Novosibirsk, Russian Federation 630117

**Keywords:** Occupational, Professional efficacy, Quality of life, Sex hormones

## Abstract

**Background:**

Very little is known about the biologic predictors of the occupational burnout in firefighters. The aim of this study was to characterize testosterone profile of active firefighters and quantify its association with three domains of the occupational burnout.

**Methods:**

We enrolled 100 firefighters (median age 28 (interquartile range (IQR) 9.8) years with 5 (IQR 9) years in service) of three fire departments in Almaty, Kazakhstan. Demographics, smoking status, health-related quality of life (HRQL) and burnout scores of Maslach Burnout Inventory were assessed using a questionnaire, while total blood testosterone was measured in venous blood. Logistic regression models were used to quantify the association of blood testosterone with each burnout domain in the adjusted for confounders models.

**Results:**

The median blood testosterone level was 14 (IQR 3.5) nmol/l and was only predicted by age (beta − 0.14, *p* < 0.01, 79% power). There were no differences in blood testosterone levels between occupational groups (Group 1 (firefighters), 14.6 (IQR 3.4); Group 2 (fire truck drivers), 14.7 (IQR 5.6); Group 3 (shift commanders, division heads, department managers and engineers), 14 (IQR 4.1) nmol/l, Kruskal-Wallis *p* = 0.32) or departments. Testosterone could not predict EX or CY, but had a negative association with PE score reflecting more burnout (odds ratio 1.18 (95% confidence interval 1.01;1.38)), adjusted for age, mental component of HRQL and education.

**Conclusions:**

Firefighters with higher testosterone may develop burnout in PE earlier, and this should be considered for proper work placement within the rescue system.

## Background

Occupational burnout remains a significant concern in almost all occupations and industries due to reduced work productivity. A recent panel of specialists working with burnout has concluded that “In a worker, occupational burnout or occupational physical and emotional exhaustion state is an exhaustion due to prolonged exposure to work-related problems” [1]. Occupational burnout is usually assessed with various tools, but none seems to offer a comprehensive understanding of burnout, because job tasks and demands contrast greatly across occupations. Nevertheless, preventing occupational burnout and understanding its triggers should be prioritized in occupations known to have higher burnout rates, such as teaching, medical staff and responders.

Firefighting is a high-risk occupation with usually high job demands, high levels of stress and the need to respond immediately. Taken together, these stressors may predispose firefighters for accelerated occupational burnout, which has been shown to be more pronounced in the managers compared to regular firefighters and fire truck drivers [2]. Predictors of accelerated burnout in firefighters are poorly understood, but perceived work stress, work-family conflict [3], effort-reward balance [4], health-related quality of life (HRQL), education, male sex and even subjective discomfort from the uniform [2] may explain occupational burnout depending on the burnout domain.

We have demonstrated earlier that male sex may yield greater burnout in professional efficacy domain, even when controlled for education level and position [2]. Given that the association of testosterone with response to stress had contradictory results in various occupations, mostly in medical workers, and that testosterone was never studied in firefighters as a predictor of any domain of burnout, we hypothesized that greater burnout in men in firefighting may be associated with testosterone levels. This is particularly relevant because testosterone can affect mood [5], quality of life [6], perceived masculinity and dominance [7]. Therefore, the aim of this presentation was to characterize testosterone profile of active firefighters and quantify its association with three domains (emotional exhaustion, cynicism and professional efficacy) of the occupational burnout.

## Materials and methods

### Study venues and design

This was a cross-sectional study in three fire departments located in Almaty, Kazakhstan. These included department, #1, #2 and #6, all located in various parts of the city, and the choice of department was stipulated by the management of the Department of Emergency Situations of Almaty. All three participating departments are listed as frequently attending to the scene of fire, the busiest in the city and fully equipped for all severity fires. Each department comprises four shifts with commanders, four division heads, firefighters, senior firefighters, drivers, driver instructors, engineers, respiratory equipment engineers and ancillary personnel. Because in the local system firefighters do not drive a fire truck, a group attending the scene of a fire consists of firefighters and drivers along with division heads and shift commanders. Each department employs more than 50 active staff.

Firefighters are in military service and work 24-h shifts with 2 days off. On days off they are still on call in case of severe emergency in the city. We divided subjects into three occupational groups: (1) firefighters and senior firefighters; (2) fire truck drivers, driver instructors and senior driver instructors; (3) management and engineers. Division heads, shift commanders, vice department heads and department supervisors made the management group. We enrolled 53 servicemen from the department #1; 40 men from the department #2 and 7 more men from the department 6. Group 1 included 49 firefighters; Group 2 consisted of 22 drivers and their instructors; Group 3 comprised 29 managers and engineers.

### Questionnaire

In each included department, we offered a structured questionnaire in Russian or Kazakh. All subjects filled the questionnaire in their workplace at the start of 24-h shift. The questionnaire was- self-administered and was offered once the informed consent was signed. It consisted of demographic part, including date of birth, sex, followed by the occupational history (current position, years in service in firefighting, department number), one question on regular exercise and a section on detailed cigarette, waterpipe and electronic cigarette smoking status. This included smoking intensity and smoking duration in years. Additionally, we asked whether study participants were on treatment with any sex hormone or their so-called boosters as sport supplements at any time during one year prior to the study. We then offered a 16-item Maslach Burnout Inventory (MBI) General (MBI-GS) to quantify occupational burnout. The mean score for each of three dimensions, emotional exhaustion (EX), cynicism (CY) and professional efficacy (PE) was calculated. The first two dimensions contained 5 items, whereas six items contributed to PE; therefore, EX and CY ranged from 0 to 5, as opposed to PE (0–6). Higher EX and CY scores are indicative of greater burnout. Higher PE score reflects lower level of burnout.

For HRQL, we applied the simplest 8-item validated SF-8 general HRQL tool and calculated the scores for the domains of physical activity (PA), social activity (SA), role physical (RP), role emotional (RE), mental health (MH), vitality (VT), bodily pain (BP) and general health (GH). We also calculated mental component score (MCS) and physical component score (PCS) as summative estimates of two domains of HRQL, which were analyzed along with the main 8 domains of the tool.

Guided by our previous report, we also included two additional questions on subjective discomfort from the uniform at work (yes/no), and difficulties in understanding the colleagues speaking the alternative language (yes/no), as these were shown to predict EX and CY, respectively.

### Testosterone measurement

Venous blood for the total testosterone was drawn from the cubital vein of a subject after the questionnaire completion at approximately 8 am in a specially designated room. All subjects were informed to abstain from alcohol for three days prior to the inclusion. We also asked firefighters to refrain from smoking 2 h prior to blood test. Four to five ml of venous blood were collected from the cubital vein into a tube and transported to a lab within one hour. Daily calibrated Access-2 (Beckman Coulter) automatic analyzer of the outside commercial laboratory was used to measure total testosterone, and the results were reported in nmol/l. Reference values for men 20 to 39 years old were 9–38 nmol/l; 40–55 years old – 6.9-21 nmol/l; above 55 years – 5.9-18.1 nmol/l.

### Statistical analysis

All variables were tested for normality and found not to be normally distributed. Therefore, we only used non-parametric tests in this analysis. The primary end-point were burnout score in each of three domains. These were analyzed both as continuous and binary variables. In the latter, we stratified patients into a high-burnout group with the MBI score above median, and a low-burnout group with the score below or equal to the median. In testing selected variables as predictors of high burnout, we used bivariate comparisons of the medians in case of continuous variable or N (%) with χ^2^ in contingency tables in case of binary predictors. Statistical significance of the probability was calculated using Mann-Whitney U- test or χ^2^ for dichotomous variables. When three or more groups were compared, we applied Kruskall-Wallis test.

All selected predictors in such bivariate analysis with *p* < 0.10 were then tested in logistic regression models, in which burnout scores were binary variables coded 0 for low burnout and coded 1 for high burnout values. At the screening stage, we assessed multicollinearity of predictors and found age and work duration to have high variance inflation factor, indicative of collinearity. Therefore, work duration was excluded from the analyses. Crude models tested one predictor, whereas adjusted analyses included all predictors of a given model, adjusting for all of them. In these models, all predictors, except department were treated as fixed effects. In all logistic regression models, we report the odds ratios (OR) with their corresponding 95% confidence intervals (CI). Tables with bivariate comparisons report medians with their interquartile ranges (IQR) or N with percent for the group. We ran all tests in NCSS 2020 (Utah, USA).

## Results

One-hundred employees from three groups were included and provided filled questionnaires and blood samples for the analysis. The median age of firefighters in the study was 28 (IQR 9.8) years with 5 (IQR 9) years in service (Table 1). Age and work duration highly correlated (Pearson r = 0.90). Subjects of Group 3, mostly on managerial positions were significantly older compared to other groups. Sixty-seven percent of employees were married with significant difference between the groups, when fewer married firefighters we in Group 1. With regard to smoking, cigarettes were most preferred tobacco products, and 36% of the staff were current smokers, as opposed to only 13% waterpipe users and 6% of those using electronic cigarettes.

**Table 1:** Basic demographic and smoking profile of included subjects

	Overall	Group 1	Group 2	Group 3	р
N (%)	100 (100)	49 (49)	22 (22)	29 (29)	–
Age, years	28 (9.8)	26 (5.5)	27 (11.8)	35 (10.5)	< 0.001
Years in service, years	5 (9)	4 (2)	5 (10.5)	12 (9.5)	< 0.001
Marital status, N (%)					
Single	31 (31)	21 (43)	7 (32)	3 (10)	< 0.001
Married	67 (67)	26 (53)	15 (78)	26 (90)	
Divorced	2 (2)	2 (4)	0 (0)	0 (0)	
Education, N (%)					
Secondary school	2 (2)	1 (2)	1 (5)	0 (0)	0.02
High school	32 (32)	13 (26)	6 (27)	13 (45)	
College	35 (35)	20 (41)	12 (55)	3 (10)	
University	31 (31)	15 (31)	3 (13)	13 (45)	
Cigarette smoking, N (%)					
Never	39 (39)	22 (44)	5 (22)	12 (41)	0.05
Former	25 (25)	13 (27)	3 (14)	9 (31)	
Current	36 (36)	14 (29)	14 (64)	8 (28)	
Electronic cigarette use, N (%)	6 (6)	1 (2)	3 (14)	2 (7)	0.16
Waterpipe smoking, N (%)	13 (13)	6 (12)	3 (14)	4 (14)	0.98

Note: means are medians with the corresponding interquartile range. Statistical significance from χ^2^ test in 2*2, 2*3 or 2*4 tables. Group 1 are firefighters and senior firefighters; Group 2 are drivers, driver instructors and senior driver instructors; Group 3 are engineers, senior engineers, shift commanders, division heads, department managers and department vice managers

One firefighter (1%) used testosterone enanthate and testosterone herbal booster sport supplement to gain more mass during his resistance training, but discontinued the use ten months prior to this study. The median blood testosterone level was 14 (IQR 3.5) nmol/l, ranging from 9.8 to 29.4 nmol/l. Among the studied demographic and other predictors, like smoking, exercise and HRQL, testosterone was only predicted by age (beta − 0.14, *p* < 0.01, 79% power). There were no subjects with abnormal testosterone levels and there were no differences in blood testosterone levels between occupational groups (Group 1, 14.6 (IQR 3.4); Group 2, 14.7 (IQR 5.6); Group 3, 14 (IQR 4.1) nmol/l, Kruskal-Wallis *p* = 0.32) or departments (*p* = 0.25). Of note, the median age in three included department did not differ either.

The overall median burnout scores were very low in the studied cohort (EX, 0.2 (IQR 0.8); CY, 0.7 (IQR 1.6); PE, 5.2 (IQR 1.7). EX scores were significantly lower (median 0 (IQR 0.4)) in the department #2 compared to department #1 (median 0.4 (IQR 1.1)) and #6 (median 0.2 (IQR 0.8)). CY and PE were, however, no different between the departments. When stratified into those with high vs. low burnout, based on the median, higher EX was associated with most HRQL domains, including PCS and MCS, and subjective discomfort from the uniform (Table 2). High CY scores were associated with cigarette smoking and MCS only. Being married or divorced, not having a university degree and having lower MCS score predicted higher PE in such analysis.

**Table 2:** The association of tested predictors with three domains of burnout

Predictor	EX		CY		PE	
	low	high	low	high	low	high
Age, years	28 (5)	28 (12.5)	27 (5.5)	29 (14)	28 (5)	28 (12)
Work duration, years	5 (6.5)	5 (11)	5 (7.3)	5 (10.3)	5 (5)	5 (11)
Testosterone, nmol/l	14 (3.2)	13.6 (5.4)	14.1 (3.5)	13.5 (4.1)	14.0 (2.9)	14.0 (5.4)
Daily cigarette smokers, N (%)	16 (29)	20 (45)	12 (24)	24 (48)*	12 (27)	24 (43)
Single, N (%)	18 (32)	13 (30)	16 (32)	15 (30)	18 (41)	12 (21)*
University degree, N (%)	17 (30)	14 (32)	16 (32)	15 (30)	19 (43)	12 (21)*
HRQL						
PA	54.1 (0)	54.1 (5.8)*	54.1 (0)	54.0 (5.8)	54.1 (0)	54.0 (4.3)
SA	55.3 (0)	55.3 (5.8)*	55.3 (0)	55.3 (0)	55.3 (0)	55.3 (0)
RP	54.0 (0)	54.0 (5.3)*	54.0 (0)	54.0 (0)	54.0 (0)	54.0 (0)
RE	52.4 (0)	52.4 (6.7)*	52.4 (0)	52.4 (1.7)	52.4 (0)	52.4 (0)
MH	56.8 (0)	56.8 (7.2)*	56.8 (0)	56.8 (7.2)	56.8 (0)	56.8 (7.2)
VT	55.6 (14.1)	55.6 (10.5)	55.6 (2.6)	55.6 (12.0)	55.6 (14.0)	55.6 (10.5)
BP	60.8 (0)	60.8 (7.4)*	60.8 (0)	60.8 (7.4)	60.8 (0)	60.8 (5.6)
GH	59.5 (6.6)	59.5 (6.6)	59.5 (6.6)	59.5 (6.6)	59.5 (6.6)	59.5 (6.6)
PCS	66.2 (2.5)	64.5 (5.4)*	66.0 (3.4)	65.8 (3.9)	66.0 (3.3)	65.4 (4.0)
MCS	67.8 (4.0)	64.7 (6.7)*	66.7 (3.8)	65.5 (7.5)*	67.6 (4.2)	65.1 (7.0)*
Language barrier	0 (0)	2 (5)	0 (0)	2 (4)	1 (2)	1 (2)
Uniform discomfort	1 (2)	6 (14)*	1 (2)	6 (12)	4 (8)	3 (6)

Note: * - *p* < 0.05. *EX* emotional exhaustion, *CY* cynicism, *PE* professional efficacy, *PA* physical activity, *SA* social activity, *PR* role physical, *RE* role emotional, *MH* mental health, *VT* vitality, *BP* bodily pain, *GH* general health, *PCS* mental component score, *MCS* mental component score

In further analyses, we tested the association of selected predictors with high burnout and found that predictors were not similar for each of included three domains of burnout (Table 3). Thus, lower EX was associated with working in department #2 and higher MCS. High CY burnout was only associated with smoking, whereas high PE burnout was associated with no university degree and greater blood testosterone. In an adjusted for MCS, age and education analysis, the OR of high burnout in PE for testosterone was 1.18 (95% CI 1.01;1.38). In a sensitivity analysis of younger fighters only (below the median age), the effect of testosterone on PE burnout was similar, OR 1.17, but wider and non-significant 95% CI.

**Table 3:** The odds ratios with their 95% confidence intervals in crude and adjusted models of selected predictors

Predictor	High EX		High CY	High PE	
	crude	adjusted	crude	crude	adjusted
Age	–	–	–	NS	NS
Testosterone	NS	NS	NS	NS	1.18 (1.01;1.38)
Daily cigarette smoking	–	–	2.92 (1.24; 6.86)	–	–
Single	–	–	–	NS	NS
University degree	–	–	–	0.36 (0.15;0.86)	0.33 (0.13;0.84)
PCS	NS	NS	–	–	–
MCS	0.90 (0.83;0.97)	0.91 (0.83;0.99)	NS	NS	NS
Uniform discomfort	7.15 (1.01;68.12)	NS	–	–	–
Dept #2	0.28 (0.11;0.60)	0.26 (0.11;0.61)	–	–	–

Note: NS – non-significant. Only crude model for CY was completed. EX – emotional exhaustion; CY – cynicism; PE – professional efficacy; PCS - mental component score; MCS – mental component score

## Discussion

This study in 100 firefighters from various positions in firefighting from three large municipal departments of Almaty was planned to ascertain the contribution of testosterone to any domain of burnout, since our earlier report yielded the association of sex with PE [2]. We further hypothesized that more testosterone in male firefighters may explain earlier burnout on this scale and found that in an adjusted analysis there was a negative significant association of blood testosterone level with PE score. This means that male firefighters with more testosterone may develop earlier of more burnout in PE, and this may have implications for placing employees on positions with high risk, where professional efficacy is critical to make decision and take action. In the current analysis, EX and CY were not associated with blood testosterone levels.

The evidence on predictors of burnout in firefighters is very limited. Only a few reports examine the association of work-family conflict, work stress, job demands, HRQL and basic sociodemographic determinants with burnout in firefighters. As in our previous report [2], we found that age cannot predict any domain of burnout in this occupational group. In other occupations, however, burnout reduced with age in men, but in women the association was bimodal [8]. We also found that smoking is a risk for greater burnout on CY scale. For firefighters, this is important given that smoking prevalence in this occupational group is quite high, and moreover, individual smoking is a significant contributor to exposure to CO [9]. Cigarette, waterpipe smoking and electronic cigarette use should be discouraged in this occupation not only because of the exposure to CO and other carcinogens, but also because this may slow down occupational burnout, thus increasing professional longevity.

Identification of biologic predictors of accelerated burnout is indeed important for first responders, because this may help placing people more resistant to burnout on the positions with high risk. Testosterone may be one such potential predictor, because testosterone not only regulates reproductive function, but also affects almost all aspects of male behavior [5], including mental health [6]. Studies on testosterone levels in firefighters are sporadic. There is only one report of the association of borderline-low testosterone with low left ventricular thickness from the annual screening data [10]. Other authors studied the estrogenic and antiestrogenic activity of the deposits on firefighters gear [11]. Serious lack of studies of testosterone levels in firefighters does not allow to understand how testosterone levels can affect burnout and how that can be used for screening and proper work placement.

Mechanism through which testosterone affects burnout is not known and needs to be elucidated in other future studies. Testosterone can also affect mental health and mood in men, and given that depression and posttraumatic stress disorder in firefighters may be fairly prevalent [12], and the association of more stress with lower testosterone [13], more testosterone should result in less stress and better feeling of self-efficacy in life and at work. The opposite effect observed in our analysis uncovers other effects of testosterone related to prolonged stress and possibly gradually exhausting effects of victories at work, which we could not know much in the last, when testosterone and burnout were not studied. In addition, testosterone levels observed in the current study were within the normal limits, whereas abnormal testosterone in firefighters, if any, would probably more dramatically affect the mood and mental health and even professional efficacy.

Our findings have preliminary implications to assess fitness to work, place firefighters on the most relevant positions and to plan prevention of early burnout in this high-risk population. Firefighters with higher testosterone may be efficient in taking action and fast response, can exhibit higher muscle power and better mood, but may develop burnout faster compared to their less masculine co-workers. Jobs in firefighting that require longevity in professional efficacy may be more appropriate for fighters with a university degree, in whom knowing their testosterone levels may help suspect faster burnout. Blood testosterone level is indeed not a unique predictor of burnout. Further research is needed to assess daily and weekly fluctuations of blood testosterone and other sex hormones as well as other biological candidates and their association with burnout. In addition, the current study is a pilot investigation of the role of sex hormones in burnout, which should be further validated in other occupations.

The inclusion of firefighters from various locations of the largest Kazakhstan city, employees of all firefighting positions, 100% response rate and adjustment of predictors of interest for significant demographic determinants and HRQL are the strengths of this presentation. The major limitation is cross-sectional design of the study, which hampers the ascertainment of the causality and the direction of association between the exposures and the outcome. Secondly, we only had access to fire departments with single visit and could not, therefore, monitor the progression of either burnout or testosterone with time. Thirdly, single testosterone measurement as opposed to series of data may limit the accuracy in measuring steroid profile of subjects. We could not measure the levels of perceived stress, work-family conflict and some other known predictors of burnout in our subjects in addition to what has been assessed using the current design. We did not identify firefighters with abnormally low testosterone in the current occupational cohort, and this is another limitation of this presentation, because low testosterone may be a risk factor for firefighter burnout and other negative outcomes. Finally, we could only measure total testosterone, but not its fractions, nor testosterone-to-cortisol ratio which may also limit the accuracy of our results.

## Conclusions

This study is the first report testing the association of testosterone with the occupational burnout in male firefighters. As opposed to EX and CY, burnout in PE may be affected by testosterone with some positive association of blood testosterone level and burnout in PE. These findings call to plan and implement studies with prospective observation of sex hormone profile and developing burnout in firefighters and other high-risk occupations. Identification of biological predictors of burnout, such as sex hormones, may guide future prevention added to the conventional efforts, such as work stress, work demand or work-family conflict reduction.

## Data Availability

The datasets used and/or analysed during the current study are available from the corresponding author on reasonable request.

## Abbreviations

BP: Bodily pain
CI: Confidence interval
CO: Carbon monoxide
CY: Cynicism
EX: Emotional exhaustion
GH: General health
HRQL: Health-related quality of life
IQR: Interquartile range
MBI: Maslach Burnout Inventory
MCS: Mental component score
MH: Mental health
PA: Physical activity
PCS: Physical component score
PE: Professional efficacy
RE: Role emotional
RP: Role physical
SA: Social activity
VT: Vitality role
